# Decreased *Streptococcus pneumoniae *susceptibility to oral antibiotics among children in rural Vietnam: a community study

**DOI:** 10.1186/1471-2334-10-85

**Published:** 2010-03-31

**Authors:** Nguyen Quynh Hoa, Nguyen V Trung, Mattias Larsson, Bo Eriksson, Ho D Phuc, Nguyen TK Chuc, Cecilia Stalsby Lundborg

**Affiliations:** 1 Division of Global Health (IHCAR), Department of Public Health Sciences, Karolinska Institutet, Nobels väg 9, 171 77 Stockholm, Sweden; 2 Vietnam Cuba Friendship Hospital, 37 Hai Ba Trung street, Hanoi, Vietnam; 3 Department of Medical Microbiology, Hanoi Medical University, 1 Ton That Tung street, Hanoi, Vietnam; 4 Clinical Laboratories, National Institute of Infectious and Tropical Diseases, 78 Giai Phong street, Hanoi, Vietnam; 5 Nordic School of Public Health, Box 12133 SE-402 42 Gothenburg, Sweden; 6 Department of Probability and Statistics, Institute of Mathematics, 18 Hoang Quoc Viet road, Hanoi, Vietnam; 7 Public Health Faculty, Hanoi Medical University, 1 Ton That Tung street, Hanoi, Vietnam

## Abstract

**Background:**

*Streptococcus pneumoniae *is the most significant bacterial cause of community-acquired pneumonia among children under five years worldwide. Updated resistance information of *S. pneumoniae *among children is essential to adjust the recommendations for empirical treatment of community-acquired pneumonia, which will have immense implications for local and global health. This study investigated the prevalence of antibiotic resistance in isolated strains of *S. pneumoniae *and relationship with antibiotic use and demographic factors of children under five in rural Vietnam in 2007.

**Methods:**

In Bavi district, 847 children 6 to 60 months were selected from 847 households. The main child-caregivers in the households were interviewed weekly using structured questionnaires to collect information of daily illness symptoms and drug use for the selected child over a four-week period (from March through June 2007). In the 3^rd ^week, the children were invited for a clinical examination and to collect nasopharyngeal samples for *S. pneumoniae *identification. Etest and disk diffusion were used to test antibiotic susceptibility.

**Results:**

Of 818 participating children, 258 (32%) had ongoing respiratory infections, 421 (52%) carried *S. pneumoniae*, and 477 (58%) had used antibiotics within the previous three weeks. Of the 421 isolates, 95% were resistant to at least one antibiotic (401/421). Resistance to co-trimoxazole, tetracycline, phenoxymethylpenicillin, erythromycin and ciprofloxacin was 78%, 75%, 75%, 70% and 28%, respectively. Low resistance was noted for amoxicillin (4%), benzylpenicillin (4%), and cefotaxime (2%). The intermediate resistance to amoxicillin was 32%. Multidrug-resistance was seen in 60%. The most common pattern was co-resistance to co-trimoxazole, tetracycline and erythromycin. The proportion of children carrying resistant bacteria was higher among the children who had used antibiotics in the previous three weeks.

**Conclusions:**

Resistance to commonly used antibiotics and multidrug-resistance of *S. pneumoniae *in the area is remarkably high. High-dose amoxicillin is the only investigated oral antibiotic that can possibly be used for treatment of community-acquired pneumococcal infections. Strategies to promote appropriate prescribing and dispensing of effective antibiotics should be immediately implemented for the benefit of local and global health.

## Background

*Streptococcus pneumoniae *is the most significant bacterial cause of community-acquired pneumonia among children under five years worldwide [1]. *S. pneumoniae *often colonizes the nasopharynx of healthy children and can then, in specific situations, spread to the lungs, paranasal tissues and cause mucosal infections, such as pneumonia, or invade the bloodstream and cause meningitis [2]. The infections caused by this pathogen are among those least likely to be resolved without effective antibiotic treatment. However, since 1980s, a dramatic increase in antibiotic resistance among *S. pneumoniae *has been observed in many parts of the world [3-7]. Treatment failure associated with antibiotic-resistant pneumococci has been reported for patients with pneumonia [7,8]. High level of antibiotic use is probably the main factor driving the emergence of resistance [9].

As in many other countries, a high prevalence of *S. pneumoniae *resistant to common antibiotics has been seen in studies in Vietnam [10,11]. A study conducted in Bavi district in 1999 showed that 90% of 106 *S. pneumoniae *isolates in 200 children were resistant to at least one antibiotic, 88% to tetracycline, 32% to trimethoprim/sulphonamide and 25% to chloramphenicol [10]. Updated resistance information, especially the resistance of *S. pneumoniae *among children, is essential to adjust the recommendations for empirical treatment of community-acquired pneumonia, which will have immense implications for local and global health.

This study is part of a larger project conducted among the population of a rural area in northern Vietnam where data on health care providers and consumers' knowledge as well as their use of antibiotics has been obtained [12,13]. The aim of this paper is to estimate the prevalence of *S. pneumoniae *carriage, to assess its antibiotic susceptibility, and to investigate possible associations to antibiotic use, demographic factors of children aged 6 to 60 months and households' economic status.

## Methods

### Study area

The study setting was Bavi district, 60 km west of Hanoi, the capital of Vietnam. The district is divided into lowland, highland and mountains. The population is approximately 262,000 persons, of whom 8% are children under five years of age. The basic public health care system includes a district hospital with 150 beds, 3 regional polyclinics and 32 commune health stations. An Epidemiological Field Laboratory (Filabavi) was established in 1998 [14]. Baseline and re-census surveys have been done every second year. Regular follow-up of vital events is conducted every three months. The surveillance database includes 69 clusters with about 51,000 inhabitants in 12,000 households. A cluster was defined as an administrative unit, usually a village. On average, there are about 600-700 inhabitants in each cluster.

### Subject and sample size

A sample of 847 children born from June 2002 to October 2006 was obtained from 847 households with at least one child eligible for the study. The sample size calculation was based on the assumption that antibiotic use in children under five in the community during one month was 70% [10], the expected drop out 30% and the design effect 2.0 due to clustering. Children older than 6 months were included to facilitate taking nasopharyngeal samples. All the households in the largest clusters in each of three geographical areas were selected to give about 280 households in each area. Some clusters were excluded due to uneven economical distribution in quintiles. In each household, one child aged 6-60 months was randomly selected (if more than one such child was present). Participants' characteristics, date of birth, sex, residential areas, parents' education, and households' assets, expenditure, and income were extracted from *FilaBavi*'s re-census survey 2007.

### Drug use survey and identification of *S. pneumoniae *carrier

The main child-caregivers in the households were interviewed weekly using structured questionnaires to collect information of daily illness symptoms and drug use for the selected child over a four-week period (from March through June 2007). The caregivers were also requested to fill a form regarding daily drug use for their child. When available, the drug package or prescription was examined by the interviewers. Drugs use for the participating children were classified according to the Anatomical Therapeutic Chemical (ATC) classification system [15], with the help of VN-pharmacy software [16]. This study includes antibiotics that are classified as antibacterials for systemic use and aggregated at the level of the active ingredient (level 5 of the ATC class J01) [15].

On the third Saturday of the survey period, the children were invited to the health commune station for a clinical examination by paediatricians from district or central hospitals. Trained microbiologists collected nasopharyngeal samples. The swabs were immediately placed in a transport tube with charcoal transport medium. Specimens were transported to Clinical Laboratories of National Institute of Infectious and Tropical Diseases, Hanoi, within 12 hours after sampling.

*S. pneumoniae *was isolated and identified using standard laboratory procedures [2,17]. Briefly, presumptive *S. pneumoniae *isolates were picked based on typical colony morphology, α-hemolysis, and Gram stain morphology. Identification was confirmed by optochin sensitivity with an inhibition zone diameter of ≥ 14 mm. A single colony of each target bacterium was selected and sub-cultured for purity check and further diagnostic measures of *S. pneumoniae*.

### Antibacterial susceptibility testing

For all isolates, minimum inhibitory concentrations (MICs) were determined for benzylpenicilin and cefotaxime using Etest (AB bioMérieux, Solna, Sweden, formerly AB BIODISK). Inhibitory zone diameters were estimated for erythromycin, co-trimoxazole, tetracycline, and ciprofloxacin using disk diffusion (BIO-RAD Laboratories, Marnes-la-Coquette, France). The tested antibiotics were those commonly used in the empirical treatment of pneumonia in Vietnam or recommended for testing by the Clinical and Laboratory Standard Institute (CLSI) [18]. Susceptibility testing was done according to the performance standards of CLSI [18] and manufacturers' instructions. *S. pneumoniae *ATCC 49619 were included as a control strain.

Interpretative non-meningitis breakpoints were based on the CLSI standards [18]. The inhibitory zone diameters for isolates to be considered resistant were: tetracycline ≤ 18 mm, co-trimoxazole ≤ 15 mm, erythromycin ≤ 15 mm. For ciprofloxacin, the criteria were: resistant ≤ 15 mm, susceptible ≥ 30 mm [19]. MICs values for cefotaxime in the Etest were: resistant ≥ 4 mg/l, susceptible ≤ 1.0 mg/l.

According to CLSI guidelines, the isolates with MICs ≤ 2.0 mg/l for benzylpenicillin were considered as susceptible at doses of at least 2 million units every four hours [18]. However, the possibility to administer the doses required by CLSI is commonly not feasible in primary health care. In order to translate *in vitro *resistance for benzylpenicillin to feasible treatment recommended for patients with pneumococcal pneumonia, we modified the current CLSI breakpoints using the European Committee on Antimicrobial Susceptibility testing (EUCAST) categorization as: susceptible MICs ≤ 0.5 mg/l, intermediate 1.0 mg/l≤ MICs ≤ 4.0 mg/l, resistant MICs ≥ 8.0 mg/l [18,20]. The pneumococcal infections caused by intermediate isolates may be appropriately treated with a high dose of antibiotics [18,20].

We defined the isolates as susceptible to amoxicillin and ampicillin using the same breakpoints as for benzylpenicillin [18]. Resistance to phenoxymethylpenicillin was derived from benzylpenicillin MICs > 0.06 mg/l [20]. Multidrug resistance was defined as isolates resistant to at least three of six tested antibiotics, i.e benzylpenicilin, cefotaxime, erythromycin, co-trimoxazole, tetracycline, and ciprofloxacin. Benzylpenicillin, ampicillin, amoxicillin were grouped together as a single class. We did not include phenoxylmethylpenicillin in the multidrug-resistant analysis since its susceptibility was derived from benzylpenicillin MIC.

### Statistical analysis

Percentages of pathogen carriers and antibiotic-resistance of *S. pneumoniae *with 95% confidence interval (95% CI) were calculated to describe the nasopharyngeal carriage of bacteria among children and resistance among identified bacterium.

A wealth index defined as a combination of the information on household income, expenditure and assets was used to define the households' economic status [21]. The analysis based on this variable was initially done using quintiles. These were then dichotomized into two levels: those identified as living below average conditions and those living at average or above conditions.

Multiple regression models were used to examine the statistical associations between antibiotic resistance and the independent variables: sex (male *vs*. female), age (24-60 months *vs*. 6-23 months), household's economic-status (average or above *vs*. below average), region (highland, mountains *vs*. lowland), present ARI symptoms including any signs of respiratory infections at the time of the nasopharynx sampling (yes *vs*. no), and antibiotics use within the three weeks preceding the sample collection (yes *vs*. no).

### Ethical considerations

The ethical review board of Hanoi Medical University approved this study (No 28/HMURB, 2006). Verbal consent of all parents was sought after explanation of the purpose of the study. Confidentiality was assured and participants were informed of their right to withdraw at any time without any explanation. Children having a condition in need of medical treatment were treated and counselled by paediatricians from district or central hospitals.

## Results

### Study population and *S. pneumoniae *carriage

For the 847 selected children, 823 (97%) caregivers gave consent to participate in the drug use survey. Of these, 818 (99%) also consented for their child to participate in the clinical examination and collection of nasopharyngeal samples. The characteristics and current health situation of the children is presented in Table 1.

**Table 1:** Characteristics of 818 community children aged 6-60 months and estimated prevalence of *S. pneumoniae *nasopharyngeal carriage.

Characteristics		Absolute number with percentage in bracket	
		
		Participating children (% among total)	*S. pneumoniae *carrier (% within group)
**Sex**	Female	373 (46)	187 (50)
	Male	445 (54)	234 (53)
**Age**	6-23 months	367 (45)	203 (55)*
	24-60 months	451 (55)	218 (48)*
**Region**	Lowland	268 (32)	145 (54)
	Highland	275 (34)	146 (53)
	Mountains	275 (34)	130 (47)
**Households' economic status**	Under average	358 (44)	196 (55)
	Average or upper	460 (56)	225 (49)
**ARI symptoms at the sampling time**	No presence	560 (68)	265 (47)*
	Presence	258 (32)	156 (61)*
**Other observation^§^**	No presence	650 (80)	340 (52)
	Presence	168 (20)	81 (48)
**Previous antibiotic use within 3 weeks**	No	341 (42)	183 (54)
	Yes	477 (58)	238 (50)

	Total	818 (100)	421 (52)

* Significant different between groups using χ^2 ^test (p < 0.05).

^§ ^Other observations: Non-ARI symptoms such as digestive disorder, skin rash, toothache, phymosis.

In the clinical examination, ARI symptoms were reported in 258 children (32%). The most common symptoms were mild ARIs such as cough, sore throat, stuffy nose, or runny nose. Seven children had fever > 38°C. Only one child was diagnosed as having pneumonia. Children aged 6-23 months were more likely to present ARI symptoms than children aged 24-60 months (34% vs. 29%). There were no significant differences regarding the presence of ARI symptoms between geographical areas or households' economic conditions. Non-ARI symptoms such as digestive disorder, skin rash, toothache, phymosis were reported for 20% of the children.

Use of antibiotics during the three weeks preceding the nasopharyngeal collection was reported for 58% of the children. The average antibiotic course was 3.54 days (SD: 2.7 days), median 3.0 days indicating that 42% used antibiotics in short courses (one or two days) and a few reported a long treatment time. Penicillins with extended-spectrum (ATC code: J01CA) such as ampicillin, amoxicillin were most commonly used (49%), followed by cephalosporins (J01D) 27%, sulphonamids and trimethoprim (J01E) 11%, macrolids (J01F) 10% and other antibiotics including tetracyclines (J01A) and aminoglycosids (J01G) 3%. Most of the antibiotic courses were used when nothing more than symptoms of mild ARIs were presented. Children living in the mountainous area more often used co-trimoxazole and those in the highland area more often used amoxicillin than other areas.

*S. pneumoniae *was isolated from 421 (52%) of the children. Children aged 6-23 months were more likely to carry *S. pneumoniae *than those aged 24-60 months (Table 1). Moreover, children who had ARI symptoms were more likely to carry the bacterium. There was no significant difference in carrier prevalence related to prior use of antibiotics or household's economic condition.

### *S. pneumoniae *antibiotic susceptibility

Table 2 shows the in-vitro activity of nine antibiotics against 421 isolates of *S. pneumoniae*. Ninety-five percent of isolates (401/421) were resistant to at least one of investigated antibiotics. A high level of resistance was found to co-trimoxazole, tetracycline, phenoxymethylpenicillin and erythromycin (70-78%). Low resistance was noted for benzylpenicillin (or amoxicillin and ampicillin) (4%) and cefotaxime (2%). However, the intermediate resistance to benzylpenicillin (or amoxicillin and ampicillin) was 32%. More than one-quarter of the *S. pneumoniae *isolates demonstrated resistance to ciprofloxacin. Only one isolate was susceptible to all antibiotics.

**Table 2:** Susceptibility of 421 *S. pneumoniae *isolates to antibiotic agents

Antibiotic agents	ATC code	Absolute number and percentage in bracket		
		
		Resistant (R)	Intermediate (I)	Susceptible (S)
Tetracycline	J01AA07	314 (75)	44 (10)	63 (15)
Benzylpenicillin	J01CE01	17 (4)	136 (32)	268 (64)
Phenoxymethylpenicillin*	J01CE02	315 (75)	-	106 (25)
Ampicillin**	J01CA01	17 (4)	136 (32)	268 (64)
Amoxicillin**	J01CA04	17 (4)	136 (32)	268 (64)
Cefotaxime	J01DD01	9 (2)	14 (3)	398 (95)
Co-trimoxazole	J01EE01	329 (78)	47 (11)	45 (11)
Erythromycin	J01FA01	294 (70)	52 (12)	75 (18)
Ciprofloxacin	J01MA02	119 (28)	296 (70)	6 (2)

* Susceptibility to phenoxymethylpenicillin is derived from benzylpenicillin MICs according to EUCAST documents.

** Susceptibility to ampicillin and amoxicillin are derived from benzylpenicillin MICs according to CLSI documents.

**Figure 1:**
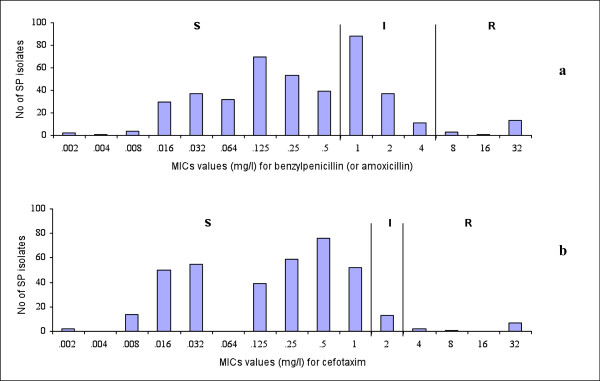
**Distribution of estimated minimum inhibitory concentration (MIC) by Etest**. Figure 1 shows the distribution of estimated minimum inhibitory concentration (MIC) for penicillin (a) and cefotaxime (b) against 421 *S. pneumoniae *isolates.

**Figure 2:**
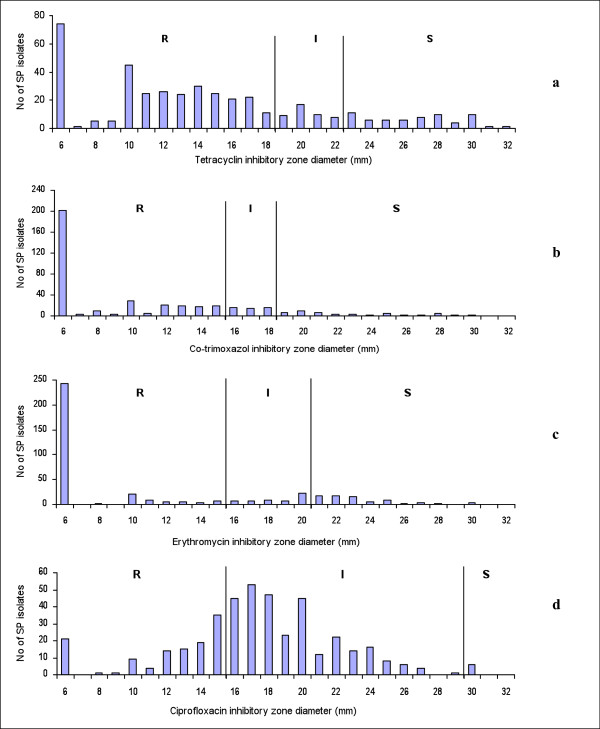
**Distribution of estimated inhibitory zone diameter by disk diffusion**. Figure 2 shows the distribution of estimated inhibitory zone diameter by disk diffusion for tetracycline (a), co-trimoxazole (b), erythoromycin (c) and ciprofloxacin (d) against 421 *S. pneumoniae *isolates.

The distribution of benzylpenicillin and cefotaxime MICs in the Etest is shown in Figure 1. The MIC of benzylpenicillin at which 90% of isolates was inhibited (MIC_90_) was 1.5 mg/l, while cefotaxime MIC_90 _was 0.75 mg/l. Moreover, we found 12 and 7 isolates for which benzylpenicillin and cefotaxime MICs were higher than 32 mg/l (Figure 1.a, b). Almost all of those isolates were resistant to co-trimoxazole, tetracycline, erythromycin, and intermediate resistant to ciprofloxacin. In the disk diffusion tests, 200/421 and 250/421 strains showed no inhibitory zone (6 mm inhibitory-zone diameter) to co-trimoxazole and erythomycin, i.e. these antibiotics had no effect on most of the isolates (Figure 2.b, c).

Table 3 shows the relation between sex, age, economic status, geographic condition, previous antibiotic use and the prevalence of antibiotic resistance. Children living in the mountainous area were more likely to carry erythromycin and cotrimoxazol-resistant isolates than those in other areas. The proportion of children carrying resistant bacteria was higher among the children who had used antibiotics within the three weeks prior to the study for all antibiotics investigated. Prior treatment of co-trimoxazole gave a higher risk of cotrimoxazol resistance of the isolates.

**Table 3:** Antibiotic resistance in relation to background characteristics of the 421 *S. pneumoniae *carriers.

Characteristics Total number of carrier in bracket	Percentage of resistant strains among total carriers within each category							
	
	TET^†^	PEN G^†^ (AMX, AMP)	PEN V^†^	CTX^†^	SXT^†^	ERY^†^	CIP^†^	MDR^†^
**Sex**								
- Female^R ^(187)	68*	5	72	3	78	67	25	56
- Male (234)	80* (**)	3	77	1	79	72	31	63
**Age**								
- 6-23 months^R ^(217)	75	4	76	3	78	73	27	61
- 24-60 months (204)	74	2	74	2	78	67	29	59
**Region**								
- Lowland^R ^(145)	77	2	78	0*	74*	68*	32	55*
- Highland (146)	72	6	77	5*	74*	64*	24	56*
- Mountains (130)	75	4	69	2*	88* (**)	78*	29	70* (**)
**Economic status**								
- Under average^R ^(203)	79	5	76	3	80	72	31	61
- Average or upper (218)	71	3	74	1	76	68	26	59
**Prior antibiotic use**								
- No^R ^(183)	73	3	68*	0.5*	73*	67	26	56
- Yes (238)	76	5	80*(**)	3*	82*(**)	72	30	63

Total (421)	75	4	75	2	78	70	28	60

* p < 0.05: significant different between groups using χ^2 ^test.

** Significant difference between group *vs*. reference using multiple logistic regression analysis.

^R ^Reference groups in multiple logistic regression analysis

^† ^TET: tetracycline, PEN G: benzylpenicillin, AMX: amoxicillin, AMP: ampicillin, PEN V: phenoxymethylpenicillin, CTX: cefotaxime, SXT: co-trimoxazole, ERY: erythromycin, CIP: ciprofloxacin, MDR: multidrug-resistant.

### Multidrug resistance among *S. pneumoniae *isolates

Most resistant isolates were multidrug resistant (252/401), and they accounted for 60% of all isolates. Isolates that were resistant to three classes of antibiotics were the most prevalent, 45% (190/421), then four antibiotics 14% (58/421). Co-resistance to five antibiotics was presented by 4 isolates.

Table 4 shows the pattern of co-resistance among the *S. pneumoniae *isolates. The most common pattern was co-resistance to co-trimoxazole, tetracycline, and erythromycin (200/252). Penicillin-resistant pneumococcal isolates are more likely to be concomitantly resistant to cefotaxime, and co-trimoxazole than to other antibiotics. Multi-resistance was not associated with sex, age, or economic status but significantly higher in children living in the mountainous area compared to other areas (Table 3).

**Table 4:** Pattern of co-resistance to tested antibiotics among 421 *S. pneumoniae *isolates

Individual antibiotic and combination	Absolute number of co-resistance with percentage of total 421 isolates in bracket							
	
		CIP^†^	PEN G^†^	CTX^†^	CIP-PEN G	CIP- CTX	PEN G-CTX	CIP- CTX - PEN G
		119 (28)	17 (4)	9 (2)	1 (0.2)*	2 (0.5)	5 (1)*	0 (0)
SXT^†^	329 (78)	86 (20)	17 (4)*	9 (2)	1 (0.2)	2 (0.5)	5 (1)	0 (0)
TET^†^	314 (75)	99 (24)*	13 (3)	7 (2)	1 (0.2)	2 (0.5)	3 (1)	0 (0)
ERY^†^	294 (70)	66 (16)*	15 (4)	6 (1)	1 (0.2)	0 (0)	4 (1)	0 (0)
SXT - TET	254 (60)*	73 (17)	13 (3)	7 (2)	1 (0.2)	2 (0.5)	3 (1)	0 (0)
SXT - ERY	248 (59)*	51 (12)	15 (4)	6 (1)	1 (0.2)	0 (0)	4 (1)	0 (0)
TET - ERY	241 (57)*	60 (14)	13 (3)	5 (1)	1 (0.2)	0 (0)	3 (1)	0 (0)
SXT-TET-ERY	200 (48)	45 (11)	13 (3)	5 (1)	1 (0.2)	0 (0)	3 (1)	0 (0)

*Significant different between resistant groups *vs*. non-resistant groups using χ^2 ^test (p < 0.05).

^† ^TET: tetracycline, PEN G: benzylpenicillin, CTX: cefotaxime, SXT: co-trimoxazole, ERY: erythromycin, CIP: ciprofloxacin.

## Discussion

This study shows that almost all of the *S. pneumoniae *isolates from the children were resistant to at least one of the investigated antibiotics. Most of the children had used antibiotics during the three weeks preceding the sample collection. There was a statistical association between antibiotic use and resistance. All the oral antibiotics investigated showed very low level of susceptibility except for high-dose amoxicillin that can possibly be used for treatment of pneumococcal infections.

*S. pneumoniae *carriage prevalence estimate was 52%, similar to the results of some other studies in Vietnam [10,11], but higher than reported elsewhere [22,23]. The carriage was lower in the older children, which is in accordance with other studies [24]. Unlike previous reports [25], however, the association between nasopharyngeal carriage of *S. pneumoniae *and prior antibacterial use was not present and this might be explained by the high levels of resistance discovered. A relationship between pneumococcal nasopharyngeal carriage to the spread of the pathogen and the development of pneumococcal diseases has been reported [26]. Implementation of conjugate vaccine could protect against pneumococcal carriage and reduce the risk of developing infection [27,28]. However, these vaccines are still expensive and may not help to reduce nasopharyngeal carriage in non-vaccine serotype carriage [27].

**Figure 3:**
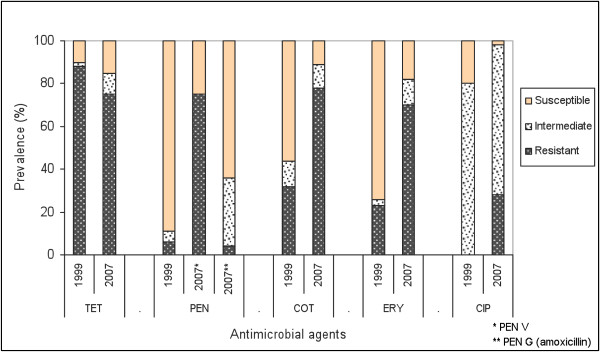
**Change of estimated susceptibility prevalence of *S. pneumoniae *isolates between 2007 and 1999**. Figure 3 compares the estimated susceptibility prevalence to five antibiotics between 421 *S. pneumoniae *isolates in 2007 and 106 isolates in 1999 in Bavi district.

The resistance to all oral commonly used antibiotics investigated among the 421 *S. pneumoniae *isolates was markedly higher than previous research [3-6]. The resistance prevalences were considerable higher than those reported in 1999 in the same area, except for tetracycline (Figure 3) [10]. A significant increase of drug-resistant pneumococcal isolates from rural area was also noted in Khanh Hoa province, Vietnam [29]. One of the most serious findings is the high multidrug resistance among the isolates (60%). This was distinctively higher than reported from the Netherlands, Russia and Sweden, however still lower than several other Asian countries [30,31]. The finding is particularly worrying, as co-resistance to co-trimoxazole-tetracycline-erythromycin is the most common pattern. The high prevalence of multi-resistant pathogens shows that the practice of combining drugs in empirical treatment in the case of resistance might not be effective in this context. Nevertheless, the national IMCI guidelines recommend five days of antibiotic therapy (anyone of co-trimoxazole, oral amoxicillin, and erythromycin) for treatment of pneumonia among children 2 months up to 5 years in the community [32]. This study shows that, co-trimoxazole and erythromycin can be expected to be virtually useless in this context and the recommendation of these drugs should be seriously considered. The emergence of *S. pneumoniae *strains showing "very high" resistance to benzylpenicillin and cefotaxime has rarely been reported internationally [33]. To address the characteristics of such isolates requires further study. The phenomenon of high resistance to commonly used antibiotics would imply a challenge for empirical treatment of pneumococcal infections in the area.

Which factors could have led to the very high level of *S. pneumoniae *antibiotic resistance and multidrug-resistance in this region? One important factor is probably the frequent and irrational use of antibiotics that constitutes a constant selective pressure. In this study area, many children frequently use antibiotics [10,34]; antibiotic use was also higher among children with pneumococcal resistant strains. The higher level of co-trimoxazole and multidrug resistance in the mountainous area may mainly be due to higher use of co-trimoxazole in this area and leading to differential selective pressure in different areas. Some resistant clones of *S. pneumoniae *spreading with different degrees of success may also be responsible for the geographical difference of resistance [35]. The easy transmission of the pathogen between individuals could facilitate the spread of multidrug resistant strains through a small number of resistant mutants [30,36]. Previous studies found two pandemic clones Taiwan 19F and Spanish 23F to be important among pneumococcal isolates in Vietnam [3,11,28,29,37]. The global spread of the pandemic multidrug-resistant serotypes possibly partly contributes to the increase in resistance in this region [37,38]. Further, the genetic determinants for resistant clones usually exist in a stable form, so once resistance is established, it is not easily lost [36]. Although the use of tetracycline in the region recently is very low compared to three decades ago, the persistence of high resistance to this antibiotic indicates the stability of the resistant clones [34]. High resistance to ciprofloxacin among the strains of children could be due to the spread of already resistant strains from adults [3,39]. The frequent inappropriate practices of prescribers and drug sellers regarding antibiotic use for treatment of acute respiratory infections may be one explanation for the high level of antibiotic use and resistance in the area [13]. Other explanation might involve patients' misperceptions and self-medication with antibiotics [12].

What antibiotics can possibly be used for empirical treatment of pneumococcal infections considering the high levels of antibiotic resistance? Clinicians may find it safe to choose susceptible antibiotics in empirical practice. On the other hand, if the effective antibiotics are more commonly used, this will promote the emergence of even more multidrug-resistant bacteria. Cefotaxime has the highest susceptibility among the investigated antibiotics. This drug is a broad-spectrum antibiotic and if used widely and indiscriminately, resistance to this antibiotic in *S. pneumoniae *may become a problem in the near future [28,40]. Hence, the still effective antibiotics need to be reserved for the severe cases in tertiary hospitals and in selected cases with troublesome resistance patterns. Benzylpenicillin has a low level of resistance (4%), but this antibiotic is administrated by injections every 4-6 hours. Therefore, it is not feasible to use benzylpenicillin as the first-line for treatment. The use of narrow spectrum penicillins such as phenoxymethylpenicillin is higher among northern Europe countries where there is also generally a low rate of antibiotic resistance [9,41]. However, high resistance to phenoxymethylpenicillin in this study (75%) may lead to a poor clinical outcome if used as empirical treatment [8]. Amoxicillin is thus the only oral antibiotic that can possibly be used for treatment of pneumococcal pneumonia among children in the study area. Being low cost, easily accessible and efficacious, amoxicillin has been strongly recommended as the initial antibiotic for treatment of non-severe pneumonia especially where co-trimoxazole resistance is common [42]. According to pharmacokinetic-pharmacodynamic principles, the appropriate dose of amoxicillin is the one that maximizes the time when the plasma concentration persists above the MICs of the etiological agent (t > MIC) [43]. For that reason, the aim is to increase the t > MIC by increasing the number of daily doses to achieve bactericidal activity against isolates with MIC ≥ 2 mg/l [18,20,44]. To assure the therapeutic effect for at least 95% of pneumococcal infections in the context, the recommended dose of amoxicillin at 25 mg/kg × 3 times daily thus appears to be too low [32,42,44]. The guidance to increase the dose needs to be further demonstrated by clinical research.

The study has several strengths. It is a community study since the children were studied in their households and not in hospitals or clinics. The latter is a common approach but the selection process will mean that the studies only represent those seeking health care. The results are internally valid for those clusters actually investigated. The generalization of the result, the external validity, must be based on theoretical propositions suggesting that clusters or district are quite similar in respects of the phenomenon under study, the antibiotic resistance. Nothing indicates that similar rural areas should be very different. The clusters selected were the largest clusters for logistic reasons. As far as it can be investigated, nothing suggests a tendency to higher carriage or resistance in the larger clusters. The participation percentage is high. The study had been carefully piloted and field-tested. The training and supervising of the interviewers and lab workers had been conducted both in the field and in the laboratory to control the quality of collected data. By using both CLSI and EUCAST guidelines to define the interpretative breakpoints for benzylpenicillin, the resistance data is considered to be more useful for developing countries' conditions.

The major limitation of the study was that due to the limited resources, we only performed Etest for benzylpenicillin and cefotaxime. The susceptibility test for ciprofloxacin is not recommended by CLSI, but the test for *S. pneumoniae *is considered as an appropriate marker for monitoring fluoroquiniolone resistance [19,20,45].

## Conclusions

Resistance to commonly used antibiotics and multidrug-resistance of *S. pneumoniae *in the investigated area are remarkably high. Of all oral antibiotics investigated, only high-dose amoxicillin can possibly be used as the first-line of choice for empirical treatment of pneumococcal infections among children in the area. The antibiotic use within the three weeks prior to the study was higher among the children with resistant pneumococci. In patients who truly warrant therapy, consideration must be given to use the most effective antibiotic class to achieve a favourable therapeutic outcome and prevent the emergence of antibiotic resistance. Strategies to promote appropriate prescribing and dispensing of effective antibiotics should be immediately implemented for the benefit of local and global health.

## Competing interests

The authors declare that they have no competing interests.

## Authors' contributions

NQH, NVT, ML, BE, HDP, NTKC, CSL participated in the conception and design of the study, and revising paper critically for substantial intellectual content. NQH supervised the data collection, performed data analysis, and drafted the manuscript. NVT was responsible for laboratory testing. NVT, ML, BE, NTKC, CSL supported in the data collection and helped to draft manuscript. BE, HDP contributed to statistical analysis. All authors read and approved the final manuscript.

## Pre-publication history

The pre-publication history for this paper can be accessed here:

http://www.biomedcentral.com/1471-2334/10/85/prepub

## References

[B1] Michelow IC Olsen K Lozano J Rollins NK Duffy LB Ziegler T Kauppila J Leinonen M McCracken GH Jr Epidemiology and clinical characteristics of community-acquired pneumonia in hospitalized children Pediatrics 2004 113 4 701 707 10.1542/peds.113.4.701 15060215

[B2] Murray PR Rosenthal KS Pfaller MA Medical Microbiology 2005 St. Louis: Elsevier Mosby

[B3] Song JH Jung SI Ko KS Kim NY Son JS Chang HH Ki HK Oh WS Suh JY Peck KR High prevalence of antimicrobial resistance among clinical Streptococcus pneumoniae isolates in Asia (an ANSORP study) Antimicrob Agents Chemother 2004 48 6 2101 2107 10.1128/AAC.48.6.2101-2107.2004 15155207 PMC415617

[B4] Adam D Global antibiotic resistance in Streptococcus pneumoniae J Antimicrob Chemother 2002 50 Suppl 1 5 12077153 10.1093/jac/dkf801

[B5] Jacobs MR Felmingham D Appelbaum PC Gruneberg RN The Alexander Project 1998-2000: susceptibility of pathogens isolated from community-acquired respiratory tract infection to commonly used antimicrobial agents J Antimicrob Chemother 2003 52 2 229 246 10.1093/jac/dkg321 12865398

[B6] Riedel S Beekmann SE Heilmann KP Richter SS Garcia-de-Lomas J Ferech M Goosens H Doern GV Antimicrobial use in Europe and antimicrobial resistance in Streptococcus pneumoniae Eur J Clin Microbiol Infect Dis 2007 26 7 485 490 10.1007/s10096-007-0321-5 17551759

[B7] Feikin DR Schuchat A Kolczak M Barrett NL Harrison LH Lefkowitz L McGeer A Farley MM Vugia DJ Lexau C Mortality from invasive pneumococcal pneumonia in the era of antibiotic resistance, 1995-1997 Am J Public Health 2000 90 2 223 229 10.2105/AJPH.90.2.223 10667183 PMC1446155

[B8] Tleyjeh IM Tlaygeh HM Hejal R Montori VM Baddour LM The impact of penicillin resistance on short-term mortality in hospitalized adults with pneumococcal pneumonia: a systematic review and meta-analysis Clin Infect Dis 2006 42 6 788 797 10.1086/500140 16477555

[B9] Goossens H Ferech M Stichele RV Elseviers M Outpatient antibiotic use in Europe and association with resistance: a cross-national database study Lancet 2005 365 9459 579 587 15708101 10.1016/S0140-6736(05)17907-0

[B10] Larsson M Kronvall G Chuc NT Karlsson I Lager F Hanh HD Tomson G Falkenberg T Antibiotic medication and bacterial resistance to antibiotics: a survey of children in a Vietnamese community Trop Med Int Health 2000 5 10 711 721 10.1046/j.1365-3156.2000.00630.x 11044266

[B11] Parry CM Diep TS Wain J Hoa NT Gainsborough M Nga D Davies C Phu NH Hien TT White NJ Nasal carriage in Vietnamese children of Streptococcus pneumoniae resistant to multiple antimicrobial agents Antimicrob Agents Chemother 2000 44 3 484 488 10.1128/AAC.44.3.484-488.2000 10681307 PMC89715

[B12] Hoa NQ Ohman A Lundborg CS Chuc NT Drug use and health-seeking behavior for childhood illness in Vietnam--a qualitative study Health Policy 2007 82 3 320 329 10.1016/j.healthpol.2006.10.005 17118482

[B13] Hoa NQ Larson M Kim Chuc NT Eriksson B Trung NV Stalsby CL Antibiotics and paediatric acute respiratory infections in rural Vietnam: health-care providers' knowledge, practical competence and reported practice Trop Med Int Health 2009 14 5 546 555 10.1111/j.1365-3156.2009.02267.x 19320870

[B14] Chuc NT Diwan V FilaBavi, a demographic surveillance site, an epidemiological field laboratory in Vietnam Scand J Public Health Suppl 2003 62 3 7 10.1080/14034950310015031 14578073

[B15] WHO collaborating Centre for Drug Statistics Methodology Anatomical Therapeutic Chemical (ATC) classification index with Defined Daily Doses (DDDs) 2009 2008 Oslo: WHO

[B16] Hanoi University of Pharmacy VN Pharmacy, the Vietnamese software for Anatomical Therapeutical Chemical Classification System with Defined Daily Dose (ATC/DDD) 2004 Hanoi: Hanoi University of Pharmacy

[B17] Michelow IC Lozano J Olsen K Goto C Rollins NK Ghaffar F Rodriguez-Cerrato V Leinonen M McCracken GH Jr Diagnosis of Streptococcus pneumoniae lower respiratory infection in hospitalized children by culture, polymerase chain reaction, serological testing, and urinary antigen detection Clin Infect Dis 2002 34 1 E1 11 10.1086/324358 11731965

[B18] Clinical and Laboratory Standards Institute (CLSI) Performance Standards for Antimicrobial Susceptibility Testing; Nineteenth Informational Supplement. M100-S19 2009 29 Wayne, USA

[B19] Zone breakpoints from SRGA & SRGA-M http://www.srga.org/

[B20] EUCAST clinical MIC Breakpoints http://www.srga.org/eucastwt/MICTAB/MICpenicillins.html

[B21] Trinh OT Nguyen ND Dibley MJ Phongsavan P Bauman AE The prevalence and correlates of physical inactivity among adults in Ho Chi Minh City BMC Public Health 2008 8 204 10.1186/1471-2458-8-204 18541020 PMC2435539

[B22] Nilsson P Laurell MH Carriage of penicillin-resistant Streptococcus pneumoniae by children in day-care centers during an intervention program in Malmo, Sweden Pediatr Infect Dis J 2001 20 12 1144 1149 10.1097/00006454-200112000-00010 11740321

[B23] Yu S Yao K Shen X Zhang W Liu X Yang Y Serogroup distribution and antimicrobial resistance of nasopharyngeal isolates of Streptococcus pneumoniae among Beijing children with upper respiratory infections (2000-2005) Eur J Clin Microbiol Infect Dis 2008 27 8 649 655 10.1007/s10096-008-0481-y 18347822

[B24] Marchisio P Esposito S Schito GC Marchese A Cavagna R Principi N Nasopharyngeal carriage of Streptococcus pneumoniae in healthy children: implications for the use of heptavalent pneumococcal conjugate vaccine Emerg Infect Dis 2002 8 5 479 484 11996682 10.3201/eid0805.010235 PMC2732490

[B25] Deeks SL Palacio R Ruvinsky R Kertesz DA Hortal M Rossi A Spika JS Di Fabio JL Risk factors and course of illness among children with invasive penicillin-resistant Streptococcus pneumoniae. The Streptococcus pneumoniae Working Group Pediatrics 1999 103 2 409 413 10.1542/peds.103.2.409 9925833

[B26] Henriques-Normark B Blomberg C Dagerhamn J Battig P Normark S The rise and fall of bacterial clones: Streptococcus pneumoniae Nat Rev Microbiol 2008 6 11 827 837 10.1038/nrmicro2011 18923410

[B27] Dinleyici EC Yargic ZA Pneumococcal conjugated vaccines: impact of PCV-7 and new achievements in the postvaccine era Expert Rev Vaccines 2008 7 9 1367 1394 10.1586/14760584.7.9.1367 18980540

[B28] Bogaert D Ha NT Sluijter M Lemmens N De Groot R Hermans PW Molecular epidemiology of pneumococcal carriage among children with upper respiratory tract infections in Hanoi, Vietnam J Clin Microbiol 2002 40 11 3903 3908 10.1128/JCM.40.11.3903-3908.2002 12409349 PMC139650

[B29] Schultsz C Vien le M Campbell JI Chau NV Diep TS Hoang NV Nga TT Savelkoul P Stepnieuwska K Parry C Changes in the nasal carriage of drug-resistant Streptococcus pneumoniae in urban and rural Vietnamese schoolchildren Trans R Soc Trop Med Hyg 2007 101 5 484 492 10.1016/j.trstmh.2006.08.010 17113613

[B30] Reinert RR Resistance phenotypes and multi-drug resistance in Streptococcus pneumoniae (PROTEKT years 1-3 [1999-2001]) J Chemother 2004 16 Suppl 6 35 48 15690684 10.1080/1120009x.2004.11782401

[B31] Chen R Chen Y Black S Hao CL Ding YF Zhang T Zhao GM Antibiotic Resistance Patterns and Serotype Distribution in Streptococcus pneumoniae from Hospitalized Pediatric Patients with Respiratory Infections in Suzhou, China J Trop Pediatr 2009 in press 19748932 10.1093/tropej/fmp078

[B32] MOH Handbook of integrated management for common childhood illness 2006 Hanoi: Ministry of Health (MOH)- Vietnam

[B33] Soriano F Cafini F Aguilar L Tarrago D Alou L Gimenez MJ Gracia M Ponte MC Leu D Pana M Breakthrough in penicillin resistance? Streptococcus pneumoniae isolates with penicillin/cefotaxime MICs of 16 mg/L and their genotypic and geographical relatedness J Antimicrob Chemother 2008 62 6 1234 1240 10.1093/jac/dkn392 18799472

[B34] Hoan LT Chuc NT Ottosson E Allebeck P Drug use among children under 5 with respiratory illness and/or diarrhoea in a rural district of Vietnam Pharmacoepidemiol Drug Saf 2009 18 6 448 453 10.1002/pds.1730 19326362

[B35] McCormick AW Whitney CG Farley MM Lynfield R Harrison LH Bennett NM Schaffner W Reingold A Hadler J Cieslak P Geographic diversity and temporal trends of antimicrobial resistance in Streptococcus pneumoniae in the United States Nat Med 2003 9 4 424 430 10.1038/nm839 12627227

[B36] Tenover FC Mechanisms of antimicrobial resistance in bacteria Am J Med 2006 119 6 Suppl 1 S3 10 10.1016/j.amjmed.2006.03.011 16735149

[B37] Parry CM Duong NM Zhou J Mai NT Diep TS Thinh le Q Wain J Van Vinh Chau N Griffiths D Day NP Emergence in Vietnam of Streptococcus pneumoniae resistant to multiple antimicrobial agents as a result of dissemination of the multiresistant Spain(23F)-1 clone Antimicrob Agents Chemother 2002 46 11 3512 3517 10.1128/AAC.46.11.3512-3517.2002 12384358 PMC128725

[B38] Linares J Ardanuy C Pallares R Fenoll A Changes in antimicrobial resistance, serotypes, and genotypes in Streptococcus pneumoniae over a thirty-year period Clin Microbiol Infect 2010 in press 20132251 10.1111/j.1469-0691.2010.03182.x

[B39] Adam HJ Hoban DJ Gin AS Zhanel GG Association between fluoroquinolone usage and a dramatic rise in ciprofloxacin-resistant Streptococcus pneumoniae in Canada, 1997-2006 Int J Antimicrob Agents 2009 34 1 82 85 10.1016/j.ijantimicag.2009.02.002 19342204

[B40] Vila-Corcoles A Bejarano-Romero F Salsench E Ochoa-Gondar O de Diego C Gomez-Bertomeu F Raga-Luria X Cliville-Guasch X Arija V Drug-resistance in Streptococcus pneumoniae isolates among Spanish middle aged and older adults with community-acquired pneumonia BMC Infect Dis 2009 9 36 10.1186/1471-2334-9-36 19320989 PMC2667188

[B41] Goossens H Ferech M Coenen S Stephens P Comparison of outpatient systemic antibacterial use in 2004 in the United States and 27 European countries Clin Infect Dis 2007 44 8 1091 1095 10.1086/512810 17366456

[B42] Grant GB Campbell H Dowell SF Graham SM Klugman KP Mulholland EK Steinhoff M Weber MW Qazi S Recommendations for treatment of childhood non-severe pneumonia Lancet Infect Dis 2009 9 3 185 196 10.1016/S1473-3099(09)70044-1 19246022 PMC7172451

[B43] Ginsburg CM McCracken GH Jr Thomas ML Clahsen J Comparative pharmacokinetics of amoxicillin and ampicillin in infants and children Pediatrics 1979 64 5 627 631 492836

[B44] Sevillano D Aguilar L Alou L Gimenez MJ Gonzalez N Echeverria O Torrico M Valdes L Coronel P Prieto J Beta-lactam activity against penicillin-resistant Streptococcus pneumoniae strains exhibiting higher amoxicillin versus penicillin minimum inhibitory concentration values: an in vitro pharmacodynamic simulation Chemotherapy 2008 54 2 84 90 10.1159/000118659 18303256

[B45] Schurek KN Adam HJ Hoban DJ Zhanel GG Call for the international adoption of microbiological breakpoints for fluoroquinolones and Streptococcus pneumoniae Int J Antimicrob Agents 2006 28 3 266 269 10.1016/j.ijantimicag.2006.04.007 16904294

